# Biosynthesis of photostable CdS quantum dots by UV-resistant psychrotolerant bacteria isolated from Union Glacier, Antarctica

**DOI:** 10.1186/s12934-024-02417-x

**Published:** 2024-05-17

**Authors:** Matías Vargas-Reyes, Nicolás Bruna, Javiera Ramos-Zúñiga, Felipe Valenzuela-Ibaceta, Paula Rivas-Álvarez, Claudio A. Navarro, José M. Pérez-Donoso

**Affiliations:** BioNanotechnology and Microbiology Laboratory, Center for Bioinformatics and Integrative Biology (CBIB), Facultad de Ciencias de la Vida, Universidad Andres Bello, Av. República # 330, Santiago, Chile

**Keywords:** Fluorescent semiconductor nanoparticles, Biological nanoparticles, Extremophilic Antarctic bacteria, Photostability

## Abstract

**Background:**

Quantum Dots (QDs) are fluorescent nanoparticles with exceptional optical and optoelectronic properties, finding widespread utility in diverse industrial applications. Presently, chemically synthesized QDs are employed in solar cells, bioimaging, and various technological domains. However, many applications demand QDs with prolonged lifespans under conditions of high-energy radiation. Over the past decade, microbial biosynthesis of nanomaterials has emerged as a sustainable and cost-effective process. In this context, the utilization of extremophile microorganisms for synthesizing QDs with unique properties has recently been reported.

**Results:**

In this study, UV-resistant bacteria were isolated from one of the most extreme environments in Antarctica, Union Glacier at the Ellsworth Mountains. Bacterial isolates, identified through 16 S sequencing, belong to the genera *Rhodococcus*, *Pseudarthrobacter*, and *Arthrobacter*. Notably, *Rhodococcus* sp. (EXRC-4 A-4), *Pseudarthrobacter* sp. (RC-2-3), and *Arthrobacter* sp. (EH-1B-1) tolerate UV-C radiation doses ≥ 120 J/m². Isolated UV-resistant bacteria biosynthesized CdS QDs with fluorescence intensities 4 to 8 times higher than those biosynthesized by *E. coli*, a mesophilic organism tolerating low doses of UV radiation. Transmission electron microscopy (TEM) analysis determined QD sizes ranging from 6 to 23 nm, and Fourier-transform infrared (FTIR) analysis demonstrated the presence of biomolecules. QDs produced by UV-resistant Antarctic bacteria exhibit high photostability after exposure to UV-B radiation, particularly in comparison to those biosynthesized by *E. coli*. Interestingly, red fluorescence-emitting QDs biosynthesized by *Rhodococcus* sp. (EXRC-4 A-4) and *Arthrobacter* sp. (EH-1B-1) increased their fluorescence emission after irradiation. Analysis of methylene blue degradation after exposure to irradiated QDs biosynthesized by UV-resistant bacteria, indicates that the QDs transfer their electrons to O_2_ for the formation of reactive oxygen species (ROS) at different levels.

**Conclusions:**

UV-resistant Antarctic bacteria represent a novel alternative for the sustainable generation of nanostructures with increased radiation tolerance—two characteristics favoring their potential application in technologies requiring continuous exposure to high-energy radiation.

**Graphical abstract:**

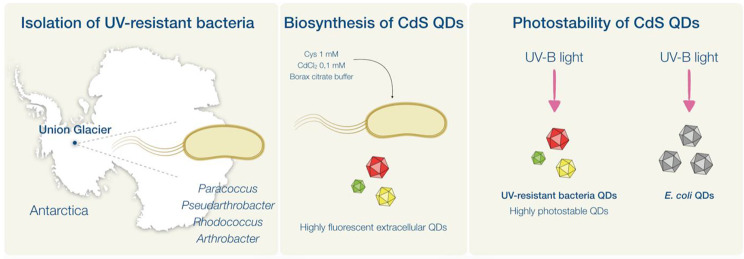

**Supplementary Information:**

The online version contains supplementary material available at 10.1186/s12934-024-02417-x.

## Background

Fluorescent semiconductor nanocrystals, commonly known as Quantum Dots (QDs), constitute nanostructures composed of elements such as Cd, Se, Te, and S. Capitalizing on the quantum confinement effect, QDs possess the ability to absorb electromagnetic radiation and emit fluorescence of varying energy, in accordance with the shape and size of the nanocrystal [1, 2]. Distinguished by superior optical properties compared to organic fluorophores, QDs exhibit a broad absorption spectrum, tunable light emission, high fluorescence quantum yield, and enhanced resistance to chemical decomposition [3]. This makes QDs a promising alternative in various fields, including biomedicine, photovoltaics, and, more recently, space applications [1, 4].

Despite their myriad applications, the use of QDs in technologies exposed to constant high-energy radiation poses challenges. Photooxidation reactions lead to QD photobleaching, significantly diminishing the efficiency and lifespan of devices. When excited by light, electrons transitioning to the conduction band may react with oxygen molecules, resulting in reduced fluorescence emission [5]. Furthermore, in biological applications, surface photooxidation contributes to the release of potentially toxic metal ions [6]. Strategies to mitigate these issues involve coating QDs with additional elements such as CdSe/CdS, CdSe/ZnS, and CdS/ZnS [7]. However, these approaches may compromise the optical properties of QDs and escalate production costs. Consequently, there is a burgeoning interest in discovering sustainable alternatives for water-soluble QDs characterized by improved photostability, cost-effectiveness, and minimal energy requirements.

While chemical synthesis is the prevailing method for QD production, its associated high costs, use of toxic inorganic solvents, and elevated energy demands [8]. In recent years, microbial biotechnology has pioneered biological methods for nanomaterial synthesis using microorganisms [9]. Microbes have been employed for the biosynthesis of CdS, CdTe, and CdSe QDs, as well as more complex structures like CdS/CdSe core/shell QDs [10–12]. This biological approach, conducted at lower temperatures (15–37 ºC) without the need for toxic solvents, presents an environmentally friendly and cost-effective alternative to chemical synthesis [8, 13, 14]. Moreover, QDs biosynthesized by bacteria exhibit water solubility due to the presence of organic molecules like peptides, proteins, and phosphates on their surfaces [15–17].

In this context, the exploration of microorganisms for biosynthesizing new nanomaterials with novel or enhanced properties has gained momentum. While bioengineering microorganisms represent an alternative, the limited understanding of the biological synthesis process and the molecules involved has hindered widespread use. An emerging alternative involves utilizing extremophile bacteria to produce QDs with improved properties. Notably, extremophilic bacteria have been reported to biosynthesize CdS QDs with high tolerance to acidic pH [18, 19] and QDs with remarkable stability under saline conditions [20]. This enhanced stability is likely attributed to biomolecules provided by extremophilic bacteria, which foster the stability of nanocrystals in harsh environments. The biosynthesis of QDs by extremophilic microorganisms has also been explored to reduce synthesis temperatures compared to chemical methods. Notably, Antarctic microorganisms have been employed to biosynthesize QDs at low temperatures (15 ºC) by bacteria of the genus *Pseudomonas* [21]. Given these precedents, extremophilic microorganisms emerge as promising candidates for serving as biofactories for the biosynthesis of QDs with unique properties.

As discussed earlier, QDs with heightened photostability are crucial for technologies exposed to constant high-energy wavelengths. Motivated by this, we hypothesize that UV-resistant extremophilic bacteria could biosynthesize QDs with increased tolerance to high-energy radiation. UV radiation induces various damages to biological organisms, including oxidative stress and direct damage to biomolecules [22]. UV-resistant bacteria typically express various biomolecules, such as pigments and low molecular weight biomolecules containing -SH groups, to mitigate these damages [23]. Interestingly, both low molecular weight biomolecules containing -SH groups and oxidoreductases have been associated with the biological synthesis of CdS and CdSe QDs [11, 15]. Leveraging this knowledge, biomolecules from UV-resistant bacteria could confer enhanced stability to QDs, mitigating photooxidation reactions and increasing QD photostability.

Antarctica, with its harsh environmental conditions, serves as an ideal environment for studying extremophilic bacteria. Despite the challenges, many microorganisms have adapted to inhabit this continent, resulting in the presence of bacteria with unique capabilities and great biotechnological potential [24]. Located 1000 km from the South Pole, Union Glacier is a hyper-extreme zone exposed to sunlight year-round, with 24 h of light during the summer months. This exposure, compounded by low cloud cover and the albedo effect, intensifies radiation at the surface level [25]. The unique conditions of Union Glacier may drive microorganisms to develop molecular strategies, such as low molecular weight biomolecules containing -SH groups and oxidoreductases, to mitigate the effects of biomolecular damage. Given these environmental characteristics, the Union Glacier region in Antarctica serves as an ideal setting for discovering UV-resistant bacteria.

In this work, we present the isolation of UV-resistant extremophilic bacteria from the Union Glacier area capable of biosynthesizing extracellular CdS QDs with enhanced photostability and fluorescence emission compared to QDs produced by mesophilic microorganisms.

## Results

### Isolation and characterization of UV-resistant bacteria

Surface, subsurface, and deep soil samples from two Union Glacier sites, Rossman Cove and Elephant Head, were collected (Table 1). From these samples, twelve bacteria were isolated on R2A culture medium and identified taxonomically as members of the genera *Paracoccus*, *Pseudarthrobacter*, *Rhodococcus*, and *Arthrobacter* (Table 2).

**Table 1:** Description of sampling sites at Union Glacier, Antarctica

Sample	GPS	Location	Depth	Soil temperature
GUJRC-2	79º 47’ 28.8’’ S 82º 55’ 57.6’’ W	Rossman Cove	Subsurface (5 cm)	-8 ºC
GUJRC-4 A	79º 47’ 27.8’’ S 82º 55’ 46.5’’ W	Rossman Cove	Surface	-8 ºC
GUJRC-4 C	79º 47’ 27.8’’ S 82º 55’ 46.5’’ W	Rossman Cove	Deep soil (22 cm)	-8 ºC
GUJEH-1B	79º 49’ 18.7’’ S 83º 19’ 50.8’’ W	Elephant Head	Subsurface (4 cm)	-10 ºC

**Table 2:** Taxonomic identification of bacteria isolated from Union Glacier soil samples based on 16 S rRNA gene sequences

Isolation code	Genus taxonomic identification	Identity (%)		Query cover (%)	Accession
RC-2-1	*Paracoccus* sp.		97.84	99	NR_044922.1
RC-2-2	*Pseudarthrobacter* sp.		86.86	98	NR_074770.2
RC-2-3	*Pseudarthrobacter* sp.		99.21	97	NR_026192.1
EXRC-2-2	*Pseudarthrobacter* sp.		98.00	98	NR_026192.1
EXRC-2-3	*Pseudarthrobacter* sp.		98.25	97	NR_026192.1
EXRC-4 A-1	*Pseudarthrobacter* sp.		97.64	97	NR_026236.1
EXRC-4 A-4	*Rhodococcus* sp.		97.56	99	NR_116275.1
EXRC-4 C-4	*Arthrobacter* sp.		97.14	99	NR_178555.1
EH-1B-1	*Arthrobacter* sp.		99.86	96	NR_113945.1
EH-1B-2	*Arthrobacter* sp.		98.00	98	NR_042252.1

**Fig. 1:**
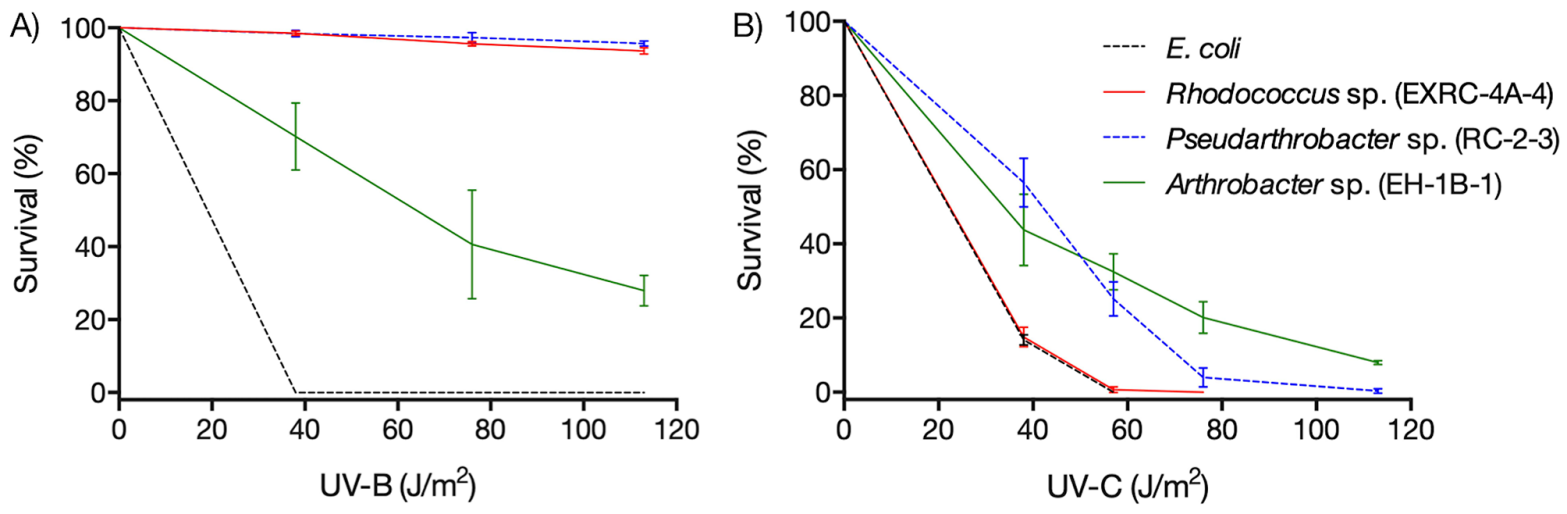
UV-B and UV-C resistance of bacterial isolates from Union Glacier, Antarctica. *Rhodococcus* sp. (EXRC-4 A-A), *Pseudarthrobacter* sp. (RC-2-3) and *Arthrobacter* sp. (EH-1B-1) were exposed to doses of 38, 57, 76, and 120 J/m^2^ of UV-B (**A**) and UV-C (**B**) radiation and the survival of cells was determined

To identify UV-resistant bacteria, isolates were exposed to UV-C radiation at a lethal dose for *E. coli* (57 J/m²). Surviving bacteria were then subjected to UV-B and UV-C doses up to 120 J/m² (Fig. 1). *Rhodococcus* sp. (EXRC-4 A-A), *Pseudarthrobacter* sp. (RC-2-3), and *Arthrobacter* sp. (EH-1B-1) demonstrated tolerance to UV-C radiation at 57 J/m². *Rhodococcus* sp. (EXRC-4 A-A) and *Pseudarthrobacter* sp. (RC-2-3) exhibited ∼ 90% survival at 120 J/m² UV-B, while *Arthrobacter* sp. (EH-1B-1) showed 28% survival at the same UV-C dose. Notably, *Arthrobacter* sp. (EH-1B-1) exhibited the highest UV-C resistance with 8% survival at 120 J/m², and *Rhodococcus* sp. (EXRC-4 A-A) and *Pseudarthrobacter* sp. (RC-2-3) demonstrated survival at UV-C doses of 60 and 80 J/m², respectively.

**Fig. 2:**
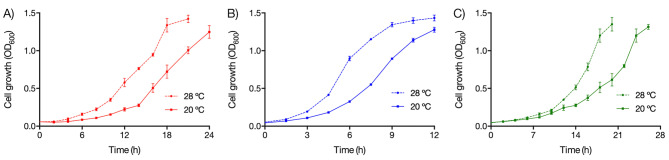
Growth of UV-resistant bacterial isolates at different temperatures. Growth curves of UV-resistant isolates *Rhodococcus* sp. (EXRC-4 A-4) (**A**), *Pseudarthrobacter* sp. (RC-2-3) (**B**), and *Arthrobacter* sp. (EH-1B-1) (**C**) were determined at 20 and 28 °C in R2A liquid medium by determining the optical density (OD_600_)

Most reported methods for biosynthesizing QDs involve bacterial cultures grown at temperatures near 37 ºC [16]. However, psychrotolerant and psychrophile Antarctic bacteria exhibit optimal growth temperatures between 20 and 28 ºC and 4 ºC, respectively [26]. To determine the optimal QD biosynthesis temperature of UV-resistant bacteria isolated from Union Glacier, we assessed the effect of temperature on bacterial growth (Fig. 2). Despite being isolated from soil samples with temperatures as low as -10 ºC (Table 1), all bacterial isolates demonstrated optimum growth at 28 ºC. Notably, the three UV-resistant bacterial isolates also displayed growth at 20 ºC, with slower growth observed at 4 ºC (data not shown), resembling the behavior of many psychrotolerant bacteria [26].

### Biosynthesis of CdS QDs by UV-resistant bacteria

**Fig. 3:**
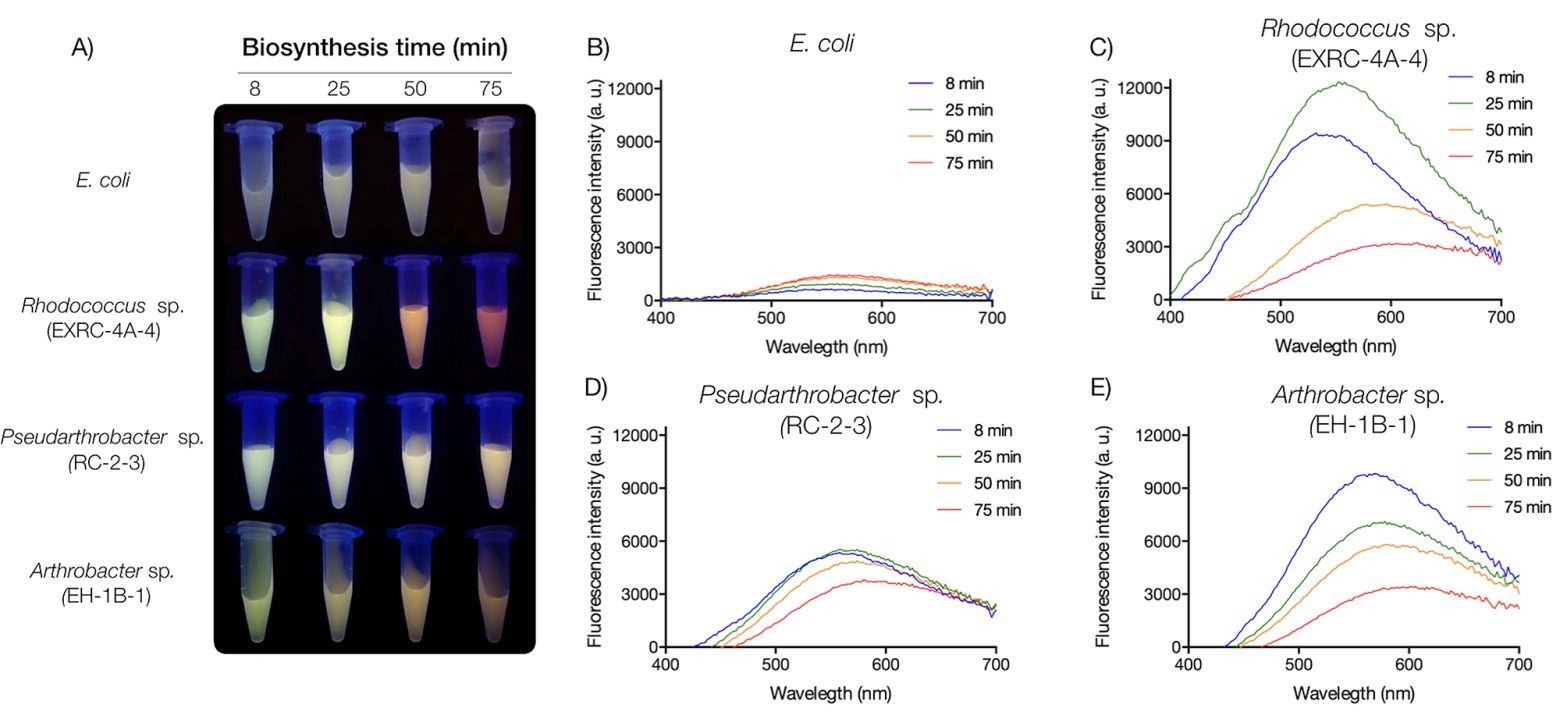
Biosynthesis of CdS QDs by UV-resistant bacteria at 20 °C. Fluorescence of biosynthesized QDs produced at 20 °C after 8, 25, 50, and 75 min synthesis reaction (**A**). Fluorescence emission spectra of QDs biosynthesized by *E. coli* (**B**), *Rhodococcus* sp. (EX-RC-4 A-4) (**C**), *Pseudarthrobacter* sp. (RC-2-3) (**D**), and *Arthrobacter* sp. (EH-1B-1) (**E**)

UV-resistant bacteria, specifically *Rhodococcus* sp. (EXRC-4 A-A), *Pseudarthrobacter* sp. (RC-2-3), and *Arthrobacter* sp. (EH-1B-1), were employed as biofactories for synthesizing CdS QDs (Fig. 3). As a control, CdS QDs were biosynthesized in *E. coli*. The biosynthesized QDs exhibited characteristic spectroscopic properties, including fluorescence emission upon UV exposure (Fig. 3A) and an absorbance spectrum (Supplementary Fig. 1). Notably, UV-resistant bacterial isolates produced CdS QDs with distinct fluorescence emissions after 8- and 75-min synthesis, featuring high fluorescence intensity (Fig. 3A, C and D, and 3E). Specifically, QDs synthesized by *Rhodococcus* sp. (EXRC-4 A-A) demonstrated the highest fluorescence intensity and a significant shift in the fluorescence emission peaks during the biosynthesis process (Fig. 3C). In addition, *Pseudarthrobacter* sp. (RC-2-3) and *Arthrobacter* sp. (EH-1B-1) displayed a minor shift in fluorescence emission peaks at 20 ºC, accompanied by elevated fluorescence intensity, particularly when compared to *E. coli* (Fig. 3D and E).

Optical, structural, and chemical properties of biosynthesized QDs were determined, including the band gap—the energy required for electrons in the valence band to reach the conduction band (measured in eV) [16]. In general, QDs biosynthesized at shorter times (8–25 min) exhibited higher Band Gap values than those biosynthesized at longer times (50–70 min) (Table 3). Furthermore, QDs produced by *E. coli* demonstrated lower Band Gap values compared to QDs synthesized by UV-resistant bacteria. This implies that QDs biosynthesized by UV-resistant bacteria require higher energy for electron movement to the conduction band but can emit a greater fluorescence intensity than QDs biosynthesized by *E. coli* (Fig. 3).

**Table 3:** Characterization of CdS QDs biosynthesized by UV-resistant bacteria and *E. coli*

Biosynthesis time (min)	FWHM (a.u.)	Band Gap (eV)	QY (%)	Size (nm)
*E. coli*				
8	187	2.82	11.3	21.4
25	167	2.82	8.7	23.5
50	185	2.70	9.2	22.7
70	174	2.80	3.8	22.6
*Rhodococcus* sp. (EXRC-4 A-4)				
8	172	2.95	12.5	5.6
25	180	2.90	12.9	8.7
50	198	2.85	7.7	10
70	216	2.82	7.6	8.7
*Pseudarthrobacter* sp. (RC-2-3)				
8	180	2.87	8.1	8.2
25	183	2.82	4.6	10.1
50	193	2.85	4.4	11.7
70	194	2.80	3.4	28.1
*Arthrobacter* sp. (EH-1-B)				
8	183	2.90	9.4	47.5
25	200	2.87	3.4	47.8
50	200	2.82	3.2	50.5
70	193	2.80	3.4	32.5

The efficiency of the fluorescence process is defined by the Quantum Yield (QY). As indicated in Table 3, CdS QDs biosynthesized by *Rhodococcus* sp. (EXRC-4 A-A) exhibited the highest QY values. These findings align with the observed fluorescence emission spectra, where CdS QDs produced by *Rhodococcus* sp. (EXRC-4 A-A) demonstrated the highest fluorescence intensity (Fig. 3). Interestingly, QDs biosynthesized by *E. coli* showed higher QY values than those produced by *Pseudarthrobacter* sp. (RC-2-3) and *Arthrobacter* sp. (EH-1B-1) (Table 3). However, as mentioned earlier, the fluorescence intensity of QDs biosynthesized by *E. coli* is lower compared to QDs synthesized by UV-resistant bacteria (Fig. 3).

**Fig. 4:**
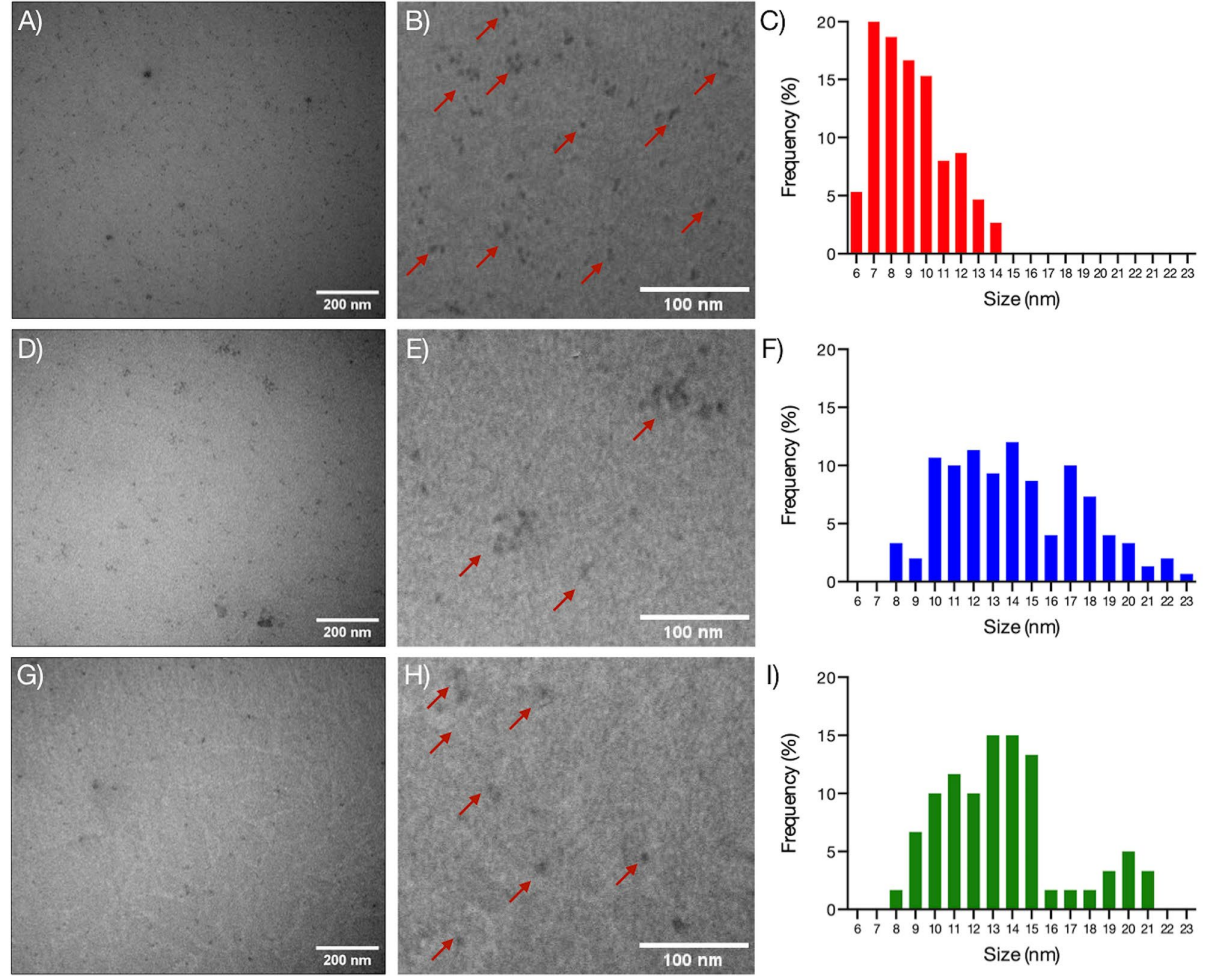
TEM analysis of CdS QDs biosynthesized by UV-resistant bacteria. Micrographs and frequency size histogram of CdS QDs biosynthesized by *Rhodococcus* sp. (EXRC-4 A-A) (**A**), *Pseudarthrobacter* sp. (RC-2-3) (**B**), and *Arthrobacter* sp. (EH-1B-1) (**C**)

To determine the size of the biosynthesized QDs, both DLS and TEM analyses were conducted (Table 3; Fig. 4). DLS measurements of the hydrodynamic radius revealed that *Rhodococcus* sp. (EXRC-4A-A) produced the smallest QDs (5.6–10 nm), followed by *Pseudarthrobacter* sp. (RC-2-3) with a range of 8.2–28.1 nm. In contrast, *E. coli* synthesized larger QDs with a range of 21.4–23.5 nm, and *Arthrobacter* sp. (EH-1B-1) produced even larger QDs ranging from 32.5 to 50.5 nm (Table 3). It is likely that QDs synthesized by *E. coli* and *Arthrobacter* sp. (EH-1B-1) contain a higher content of organic matter compared to those synthesized by *Rhodococcus* sp. (EXRC-4A-A) and *Pseudarthrobacter* sp. (RC-2-3). For a more detailed analysis of size at 25 min synthesis (yellow fluorescence emitting QDs), TEM was employed. Figure 4 displays the TEM results, indicating that QDs produced by *Rhodococcus* sp. (EXRC-4A-A) (Fig. 4A) ranged in size from 6 to 14 nm, while QDs from *Pseudarthrobacter* sp. (RC-2-3) (Fig. 4B) and *Arthrobacter* sp. (EH-1B-1) ranged from 6 to 23 nm, confirming their nanometric dimensions. As noted in previous studies on the biosynthesis of QDs, wherein QDs typically exhibit negative Z-potential values, the UV-resistant bacteria isolated from Union Glacier demonstrated negative Z-potential values of -5.93 mV (*Rhodococcus* sp. (EXRC-4A-4), -6.56 mV (*Pseudarthrobacter* sp. (RC-2-3), and − 1.79 mV (*Arthrobacter* sp. (EH-1B-1). According to Suresh et al. (2011) these negative Z-potential values suggest enhanced stability of the nanoparticles, as the electrostatic repulsive forces between the nanoparticles may prevent their association and subsequent agglomeration or clumping in aqueous suspension [27]. Consequently, these findings corroborate the results obtained in our present investigation, wherein the biosynthesized QDs exhibited remarkable photostability.

QDs were characterized for their composition using energy-dispersive X-ray spectroscopy (EDS) and for their crystal structure using X-ray diffraction (XRD) (Supplementary Fig. 2). The QDs biosynthesized by the UV-resistant bacteria were identified as CdS QDs, with an observed 1:1 ratio of Cd and S, consistent with previous findings for this type of nanoparticles (Supplementary Fig. 2A, 2B, and 2 C). Additionally, peaks corresponding to C and O were detected, indicative of organic matter present in the nanoparticle coating, along with a Na peak attributed to the Borax-citrate Buffer utilized in the biosynthesis process of QDs. Furthermore, XRD analysis confirmed the crystalline structure of the QDs, as depicted in Supplementary Fig. 2D, with peaks consistent with previously reported patterns for biological QDs, thus validating the presence of CdS QDs [28–30]. However, diffractograms obtained from biosynthesized QDs from UV-resistant bacteria revealed a predominant amorphous component. This observation is in line with existing literature documenting the tendency of CdS nanoparticles, especially when biosynthesized or functionalized with proteins, to exhibit an amorphous morphology [31]. Our current findings support and extend these observations, as evidenced by our prior analyses employing TEM, FTIR, and DLS. Collectively, these analyses reinforce the proposition that biosynthesized nanoparticles are coated with organic compounds, likely influencing their properties.

Biological QDs are characterized by an organic coating primarily composed of peptides and proteins [12]. Consequently, the organic composition of the biosynthesized QDs was analyzed using Fourier-transform infrared spectroscopy (FTIR) (Supplementary Fig. 3). The FTIR spectra of the biosynthesized QDs revealed distinctive peaks corresponding to various functional groups, including hydroxyl groups (at 3350 cm-1), C-H interactions of aliphatic carbons (at 2950 cm-1), C-O and NH groups of amines and amides (near 1590 and 1400 cm-1), and C-C double bonds (at 1590, 1400, and 970 cm-1). Consistent with prior reports [20], the FTIR analyses indicated the presence of organic matter in QDs biosynthesized by *E. coli*. Furthermore, the analyses suggested the presence of organic matter in CdS QDs biosynthesized by UV-resistant bacteria, potentially including biomolecules such as proteins.

### CdS QDs photostability

**Fig. 5:**
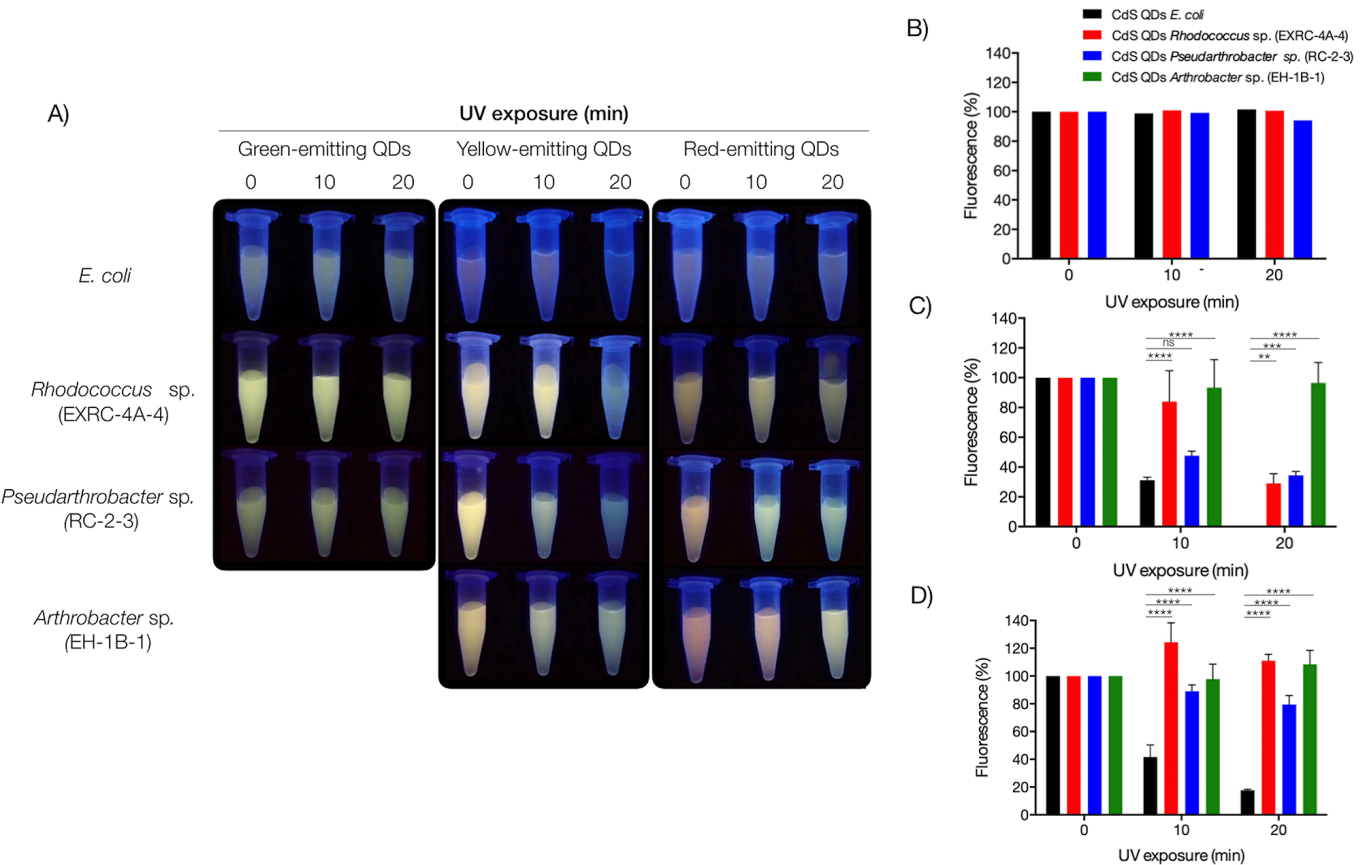
Photostability of QDs biosynthesized by UV-resistant bacteria. Green-emitting, yellow-emitting, and red-emitting QDs were exposed to UV irradiation (280–360 nm) for 10 and 20 min, and the fluorescence of tubes containing QDs solutions was determined (**A**). The percentage of fluorescence decay of green-emitting (**B**), yellow-emitting (**C**), and red-emitting QDs (**D**) was determined by measuring the fluorescence emission peak of the irradiated QDs compared to non-irradiated QDs. QDs from *E. coli*, a bacterium tolerant to low UV doses, were used as a control condition. Two-way ANOVA (*p*-value = ** 0.021; ***0.004; **** <0.001)

The photostability of CdS QDs biosynthesized by UV-resistant bacteria was systematically evaluated, focusing on QDs with different fluorescence emissions: green-emitting (< 570 nm), yellow-emitting (570–600 nm), and red-emitting QDs (> 600 nm). These QDs underwent exposure to UV radiation (280–360 nm) for durations of 10 and 20 min, and subsequent evaluation of fluorescence decay ensued. CdS QDs biosynthesized by *E. coli* were employed as a control condition. Green-emitting QDs, produced by *E. coli*, *Pseudarthrobacter* sp. (RC-2-3), and *Rhodococcus* sp. (EXRC-4 A-4), exhibited maintained fluorescence levels after irradiation (Fig. 5A). Yellow-emitting and red-emitting QDs biosynthesized by *E. coli* displayed a significant decay in fluorescence emission (Fig. 5B and C). In contrast, QDs biosynthesized by UV-resistant bacteria demonstrated remarkable photostability, maintaining their fluorescence emission after UV irradiation. Interestingly, yellow and red-emitting QDs produced by *Arthrobacter* sp. (EH-1-B) and *Rhodococcus* sp. (EXRC-4 A-4) not only maintained their fluorescence intensity but also exhibited a change in the peak wavelength of fluorescence emission after 10 and 20 min of UV exposure (Fig. 5D). Previous reports have suggested that radiation exposure can induce changes in QD size, subsequently altering the fluorescence emission wavelength [32]. To confirm this, the sizes of QDs after UV exposure were determined using DLS (data not shown). Yellow-emitting QDs biosynthesized by *Pseudarthrobacter* sp. (RC-2-3) decreased in size from 11.7 to 8.7 nm, while red-emitting QDs decreased from 28 to 2.3 nm. Similarly, yellow-emitting QDs from *Arthrobacter* sp. (EH-1-B) decreased from 50 to 13 nm, and red-emitting QDs decreased from 32 to 10 nm. This phenomenon indicates that UV radiation induces a size decrease in QDs, resulting in a change in fluorescence emission wavelength and an increase in fluorescence emission of the nanoparticles. Based on these results, it is evident that QDs biosynthesized by UV-resistant bacteria exhibit greater stability to UV radiation compared to those biosynthesized by *E. coli*. Notably, the fluorescence of yellow and red-emitting QDs produced by *Arthrobacter* sp. (EH-1-B) and *Rhodococcus* sp. (EXRC-4 A-4) even increased upon exposure to UV radiation.

**Fig. 6:**
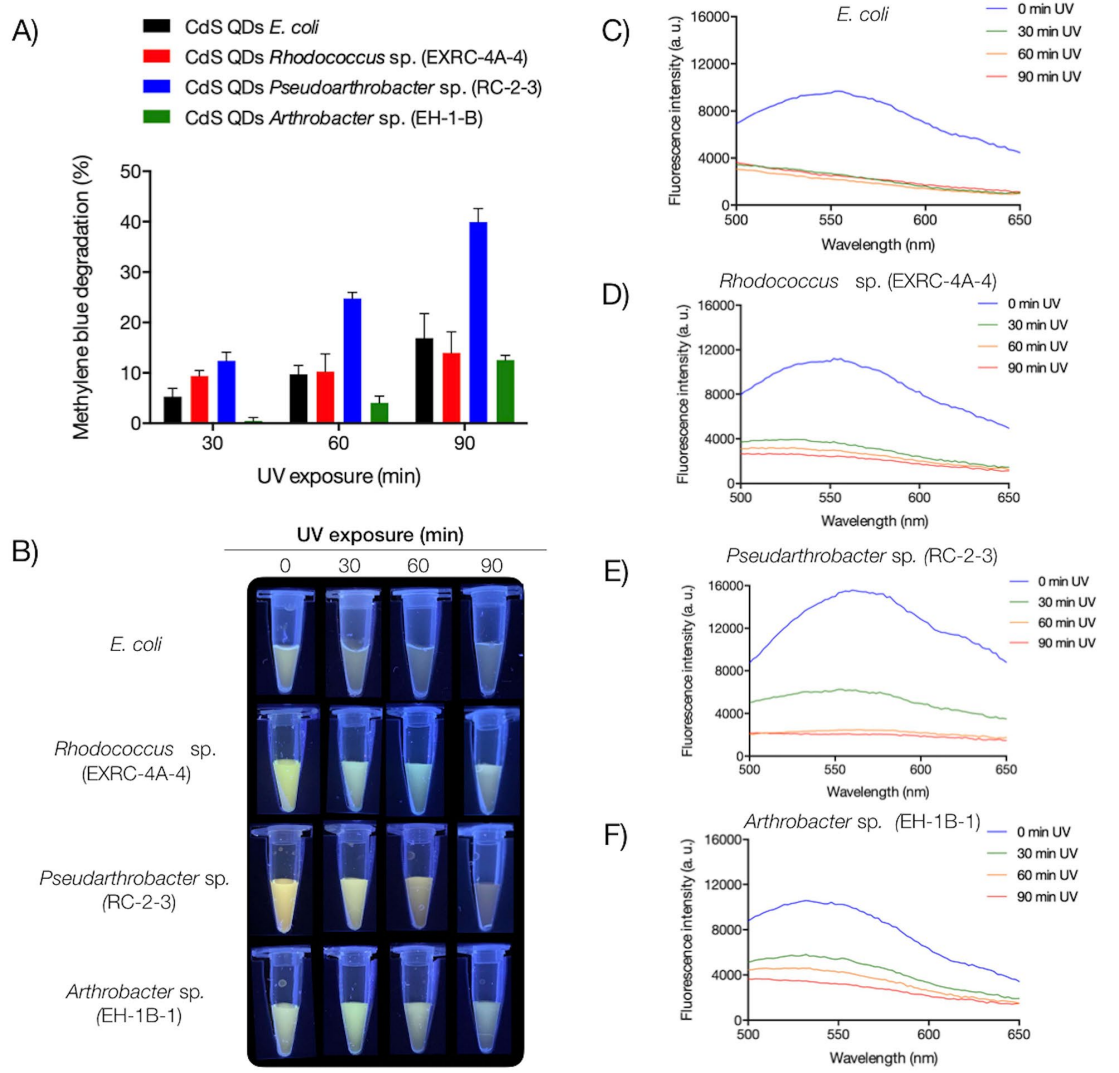
Methylene blue photocatalytic degradation mediated by biosynthesized CdS QDs. (**A**) Methylene blue degradation in presence of CdS QDs irradiated at UV (280–360 nm) during 30, 60, and 90 min. (**B**) Fluorescence of the QDs visualized after exposure to UV during 30, 60, and 90 min. Emission spectra (400 to 700 nm, 360 nm excitation) of CdS QDs produced by *E. coli* (**C**), *Rhodococcus* sp. (EXRC-4 A-4) (**D**), *Pseudarthrobacter* sp. (RC-2-3) (**E**), and *Arthrobacter* sp. (EH-1B-1) (**F**). Two-way ANOVA (*p*-value = ** 0.021; ***0.004; **** <0.001)

One reason for the decrease in the fluorescence of biosynthesized QDs upon exposure to UV radiation could be attributed to photochemical reactions, notably photobleaching. Photobleaching of QDs generally occurs due to electron transfers to molecules present in the medium [5]. QDs, when excited with wavelengths equal to or greater than their band gap, can generate an electron-hole pair. Upon interaction with molecules such as O_2_ or H_2_O, photo-electrons can produce free radicals that efficiently oxidize organic molecules [5]. To assess whether the photobleaching of QDs biosynthesized by UV-resistant bacteria results from electron loss, the QDs-mediated degradation of methylene blue was investigated (Fig. 6). Irradiation of QDs synthesized by *E. coli* led to methylene blue degradation of 5.3% at 30 min, 9.7% at 60 min, and 16.9% at 90 min (Fig. 6A). Similarly, QDs biosynthesized by *Rhodococcus* sp. (EXRC-4 A-4) caused methylene blue degradation of 9.4% at 30 min, 10.6% at 60 min, and 14.0% at 90 min. QDs from *Arthrobacter* sp. (EH-1-B) resulted in the lowest percentage of methylene blue degradation: 0.5% at 30 min, 4.1% at 60 min, and 12.6% at 90 min. QDs synthesized by *Pseudarthrobacter* sp. (RC-2-3) caused the highest percentage of methylene blue degradation, with values of 12.4%, 24.8%, and 39.9% at 30, 60, and 90 min, respectively. No methylene blue degradation was observed in the absence of light (Supplementary Fig. 4). These results suggest that the irradiation of QDs induces the transfer of electrons to oxygen, generating free radicals that oxidize methylene blue. In parallel, UV exposure of the QDs resulted in a fluorescence decrease at varying levels. UV exposure for 30 min led to the total loss of fluorescence in QDs biosynthesized by *E. coli* (Fig. 6B and C). Conversely, QDs biosynthesized by *Rhodococcus* sp. (EXRC-4 A-4) and *Arthrobacter* sp. (EH-1-B) maintained their fluorescence emission even after 90 min of UV exposure, demonstrating the high photostability of QDs biosynthesized by UV-resistant bacteria (Fig. 6B and D, and 6F). QDs from *Pseudarthrobacter* sp. (RC-2-3) lost their fluorescence emission at 60 min of UV exposure, likely due to a higher electron transfer resulting in a higher percentage of methylene blue degradation (Fig. 6A and E). Collectively, these results suggest that exposure of QDs to UV radiation induces photobleaching due to the loss of electrons from the nanoparticle core. This phenomenon leads to changes in the optical and structural properties of the QDs.

## Discussion

In the present study, we successfully isolated UV-resistant bacteria from Union Glacier in Antarctica, assessing their ability to biosynthesize photostable CdS QDs. The isolated bacterial strains were identified and classified into the *Paracoccus*, *Rhodococcus*, *Pseudarthrobacter*, and *Arthrobacter* genera (Table 2). These genera are well-documented in Antarctica, with previous reports highlighting their presence in diverse environmental niches [33, 34]. Bacteria belonging to the genus *Paracoccus* have been notably identified in nutrient-poor environments, including oceanic and Antarctic sediments [35]. The isolation of members of the *Pseudarthrobacter* genus from Antarctic soil samples aligns with previous findings, where some strains have been classified as psychrophilic bacteria, thriving in cold environments [36]. The genus *Arthrobacter*, known for its versatility, exhibits the capacity to survive in nutrient-scarce soils and endure challenging environmental conditions [37]. Genomic studies have shed light on the resilience of *Arthrobacter* bacteria, revealing an array of cold-tolerance mechanisms. These include the expression of genes associated with carotenoid biosynthesis, cold-shock proteins, and genes involved in oxidative and osmotic stress responses [38]. Such adaptations underscore the ability of these bacteria to thrive in the harsh Antarctic environment. By exploring and characterizing UV-resistant bacteria from Antarctica, our study contributes to the understanding of microbial diversity and adaptation strategies in extreme environments. In this context, while working on this project, one of the bacteria employed, *Arthrobacter* sp. (EH-1B-1), was identified as a novel species and is now designated as *Arthrobacter vasquezii* [39]. Presently, we are engaged in studies on the bacteria *Rhodococcus* sp. (EXRC-4 A-4) and *Pseudarthrobacter* sp. (RC-2-3) to determine whether they represent new species. The subsequent evaluation of their capacity to biosynthesize CdS QDs adds a novel dimension to the potential biotechnological applications of these unique bacterial isolates.

The isolates *Rhodococcus* sp. (EXRC-4 A-4), *Pseudarthrobacter* sp. (RC-2-3), and *Arthrobacter* sp. (EH-1B-1) demonstrated remarkable resistance to UV radiation, as illustrated in Fig. 1. Previous studies on microorganisms inhabiting regions with elevated radiation levels, such as the Atacama Desert or Antarctica, have reported a spectrum of UV-C tolerance. For instance, Marizcurrena et al. (2017) identified bacteria from the Antarctic Peninsula with UV-C tolerance ranging between 50 and 200 J/m^2^ [40]. Similarly, Paulino-Lima et al. (2013) described UV-C-tolerant bacteria from the Atacama Desert capable of withstanding doses up to 600 J/m^2^ [41]. The substantial UV resistance observed in the bacteria isolated in this study may be attributed to the persistent radiation exposure in the natural environment of Union Glacier. Microorganisms in high-radiation environments have evolved various mechanisms to counteract the detrimental effects of different UV wavelengths [42]. Bacterial resistance to UV radiation encompasses several strategies, including the presence of pigments, antioxidant barriers, enzymes countering the effects of oxidative stress (especially in the case of UV-B radiation), and DNA and protein repair mechanisms (particularly relevant for UV-C radiation) [22, 43, 44].

Traditional methods for the chemical synthesis of QDs often involve high temperatures (100–300 ºC), anaerobic environments, and the use of organic solvents [8]. The utilization of medium-low temperatures in QDs synthesis holds significant interest for industrial-scale production due to its potential advantages. In our study, we discovered that UV-resistant bacteria *Rhodococcus* sp. (EXRC-4 A-A), *Pseudarthrobacter* sp. (RC-2-3), and *Arthrobacter* sp. (EH-1B-1) exhibit the capability to biosynthesize fluorescent QDs at a moderate temperature of 20 ºC (Fig. 3). This finding suggests a direct correlation between the biosynthesis of QDs in bacteria and the temperature at which the microorganisms grow. Notably, UV-resistant bacteria demonstrated efficient growth at 20 °C, as illustrated in Fig. 2. Our group previously reported one of the lowest temperatures for QDs biosynthesis, achieving the production of CdS QDs at 15 °C by *Pseudomonas* sp. isolated from Antarctica [21]. While few reports have delved into the biosynthesis of QDs at low temperatures, our study contributes to this limited body of knowledge and highlights the potential for environmentally friendly and energy-efficient QDs production at medium-low temperatures.

UV-resistant bacteria *Rhodococcus* sp. (EXRC-4 A-4), *Pseudarthrobacter* sp. (RC-2-3), and *Arthrobacter* sp. (EH-1B-1) demonstrated the ability to biosynthesize extracellular CdS QDs in the presence of cysteine. Previous studies on QD biosynthesis have shown that the enzyme cysteine desulfhydrase catalyzes the production of H_2_S in the presence of cysteine, which serves as the sulfur source for CdS QDs [45]. Additionally, reports indicate that QDs biosynthesized by bacterial cells are covered with organic molecules, including proteins and peptides [17]. UV-resistant bacteria employ various mechanisms to mitigate radiation damage, utilizing antioxidant molecules such as glutathione, as well as enzymes like thioredoxins and glutaredoxins [42]. Both glutathione and thioredoxins, as well as glutaredoxins, have been detected in the organic matter covering QDs [11, 15]. Given this background, it is likely that many of the biomolecules used by cells to defend against UV stress act as nucleation centers or stabilizing agents during the process of nanocrystal formation.

QDs biosynthesized by Antarctic UV-resistant bacteria exhibit significantly higher fluorescence intensity compared to those produced by *E. coli* (Fig. 3). Notably, QDs synthesized by *Rhodococcus* sp. (EXRC-4 A-4) demonstrate the highest fluorescence intensity and QY values among the studied bacteria (Fig. 3; Table 3). Several factors could contribute to the observed increase in fluorescence. The biomolecules constituting the biosynthesized QDs, alterations in the structural properties of the QDs, or an augmented production of QDs may all play a role. Biological QDs are known to incorporate an organic capping of biomolecules, including proteins, peptides, phosphates, and thiolated molecules like glutathione [11, 15, 16]. FTIR analysis (Supplementary Fig. 3) further confirms the presence of characteristic biomolecular functional groups such as hydroxyl groups, C-H interactions of aliphatic carbons, C-O and NH of amines and amides, and C-C double bonds. The incorporation of these ligands in QDs has been reported to have direct effects on their optical properties [46]. Given that the UV-resistant bacteria utilized as QD biosynthesis platforms belong to distinct bacterial genera, namely *Rhodococcus*, *Pseudarthrobacter*, and *Arthrobacter*, the varied composition of biomolecules introduced by these bacteria may contribute to the observed increase in fluorescence emission. This diversity highlights the potential of leveraging different bacterial strains for tailoring the optical properties of biosynthesized QDs, offering avenues for fine-tuning their applications in various fields.

QDs biosynthesized by UV-resistant Antarctic bacteria exhibit superior photostability compared to those produced by *E. coli*. Notably, yellow-emitting and red-emitting QDs synthesized by UV-resistant bacteria display only a mild decrease in fluorescence emission after exposure to UV radiation, a phenomenon observed to a greater extent in QDs biosynthesized by the non-UV-resistant bacterium *E. coli*. The remarkable photostability observed in UV-resistant bacterial isolates can be attributed to their unique physicochemical properties, including size, charge, concentration, and the composition of their coating material [47, 48]. As previously mentioned, QDs synthesized by living microorganisms present a coating composed of proteins, nucleic acids, and antioxidant molecules [15]. The UV-resistant bacterial isolates employed in this study may produce biomolecules with a high potential to stabilize QDs, preventing contact with molecular oxygen that could otherwise lead to photooxidation reactions and subsequent photobleaching.

Upon exposure to UV radiation, yellow-emitting and red-emitting QDs produced by UV-resistant bacteria exhibited a wavelength shift in the fluorescence emission peak (blue shift) (Fig. 5). Additionally, DLS analysis revealed a decrease in the sizes of the QDs after irradiation. This behavior aligns with previous reports suggesting that radiation exposure induces a decrease in QD size [49, 50]. UV radiation can initiate photooxidation reactions affecting thiol groups on the QD surface, resulting in a decrease in the nanoparticle core size, potential photobleaching, and aggregation of QDs [51]. Remarkably, red-emitting QDs produced by *Rhodococcus* sp. (EXRC-4 A-4) and *Arthrobacter* sp. (EH-1-B) demonstrated an increase in fluorescence emission after irradiation. This intriguing phenomenon aligns with recent studies describing Photoinduced Fluorescence Enhancement (PFD), a behavior where the fluorescence intensity of QDs increases with irradiation time [50]. PFD occurs due to the transfer of electrons from irradiated QDs to oxygen molecules, forming superoxide radicals (^·^O_2_^−^). Surface oxidation of QDs promotes recombination through surface states, leading to an enhancement in fluorescence intensity. Notably, PFD has not been reported in QDs biosynthesized by bacteria, making this study potentially the first to describe the occurrence of PFD in biological QDs. These findings underscore the unique and advantageous photophysical properties of QDs biosynthesized by UV-resistant Antarctic bacteria. The combination of enhanced photostability and the potential occurrence of PFD opens new avenues for the application of biological QDs in various fields, highlighting their versatility and potential impact on emerging technologies.

The changes in the optical and structural properties of biosynthesized QDs exposed to UV radiation are attributed to the loss of electrons from the QDs, facilitating the formation of reactive oxygen species (ROS) that oxidize the external capping of the QDs [5, 51]. Radiation-induced electron promotion from the valence band to the conduction band generates a positively charged hole (H^+^) in the valence band. Upon the return of electrons to the valence band, energy is released as fluorescence. Notably, molecular oxygen (O_2_) can accept electrons from the conduction band, forming superoxide radicals (^·^O_2_^−^). Additionally, water (H_2_O) adsorbed on the QDs’ surface can be oxidized by the H^+^ in the valence band, generating hydroxyl radicals (^·^OH). Various methods exist for determining ROS produced by irradiated QDs, with the oxidation of organic pigments like methylene blue commonly used as an indicator [52]. In our study, biosynthesized QDs exposed to UV irradiation caused the degradation of methylene blue, signifying the transfer of excited electrons to O_2_, resulting in the generation of ^·^O_2_^−^ (Fig. 6). Notably, QDs biosynthesized by each bacterium exhibited differences in electron transfer to O_2_, evidenced by varying percentages of methylene blue degradation. Furthermore, QDs biosynthesized by UV-resistant bacteria maintained their fluorescence after irradiation, showcasing their remarkable photostability. The ability to degrade methylene blue is indicative of differences in the properties of biosynthesized QDs by each bacterium. This versatility positions UV-resistant bacteria as excellent platforms for biosynthesizing QDs with unique properties, suitable for diverse applications. For instance, *Pseudarthrobacter* sp. (RC-2-3) biosynthesized QDs exhibit high photostability and generate elevated levels of ROS, making them ideal candidates for photocatalysis applications and Quantum Dots Sensitized Solar Cells (QDSSCs) [53–55]. In contrast, *Arthrobacter* sp. (EH-1B-1) biosynthesized QDs, characterized by low ROS levels and high photostability, could find applications in constant UV light exposure scenarios, such as fluorophores in biomedicine [56, 57]. Similarly, *Rhodococcus* sp. (EXRC-4 A-4) biosynthesized QDs, with their high photostability and fluorescence emission, are well-suited for imaging applications. These findings position UV-resistant extremophilic bacteria from Union Glacier as promising platforms for the biosynthesis of QDs tailored for industrial and scientific applications.

## Conclusion

In summary, our study unveils a significant achievement in the biosynthesis of highly fluorescent and photostable QDs using UV-resistant bacteria isolated from the extreme environment of Union Glacier in Antarctica. The distinctive properties exhibited by these QDs position them as promising candidates for a wide range of applications, including photovoltaic, photocatalysis, biomedical, and beyond. This work underscores the remarkable capacity of extremophilic bacteria to serve as potent platforms to produce nanoparticles endowed with unique and advantageous characteristics. The insights gained from this study contribute to the growing field of bionanotechnology, showcasing the potential of microorganisms thriving in extreme conditions to play a pivotal role in the development of advanced nanomaterials with diverse applications.

## Methods

### Soil samples

Soil samples were collected from two distinct sites within the Ellsworth Mountains, namely Rossman Cove and Elephant Head, both situated in Union Glacier (Table 1). Subsequently, the samples were carefully stored in sterile bags to maintain their integrity until processing at the laboratory.

### Bacterial isolation from Union Glacier soil samples

To isolate bacteria from the Union Glacier soil samples, three grams of soil were suspended in 30 mL of phosphate-buffered saline (PBS) and incubated at 20 °C with stirring for a period of 2 days. Subsequently, a 30 µL aliquot of the suspension was inoculated onto R2A solid media. The R2A solid media composition consisted of 0.5 g yeast extract, 0.5 g casamino acids, 0.5 g peptone, 0.5 g glucose, 0.3 g pyruvic acid, 0.3 g K_2_HPO_4_, 0.05 g MgSO_4_ * 7H_2_O, and 20 g agar per liter. Inoculated plates were then incubated at 4, 12, and 28 °C until the growth of bacterial colonies was observed.

### Taxonomic identification of bacterial isolates

The taxonomic identification of bacterial isolates was accomplished through 16 S rRNA sequencing. DNA extraction was performed by subjecting bacterial lysates to a thermal lysis step at 95 °C for 10 min in 50 µL of nuclease-free water. After centrifugation at 14,000 RPM for 10 min, the supernatant containing the extracted DNA was utilized as a template for PCR. Amplification was carried out using universal primers 27 F (5’- AGA GTT TGA TCM TGG CTC AG -3’) and 1525R (5’- TAC GGY TAC CTT GTT ACG ACT T -3’). The resulting amplicons were subjected to agarose gel electrophoresis (1%), purified using the FavorPrepTM GEL/PCR Purification kit (Favorgen), and subsequently sequenced by Macrogen Ing. Korea. Taxonomic classification of bacterial isolates was achieved at the genus level using the BLAST algorithm with the 16 S ribosomal RNA sequences database.

### Bacterial UV-resistance screening

To assess the UV-resistance of bacterial isolates, screening was conducted by exposing the isolates on R2A solid medium to either UV-B (280–360 nm, UV Lamp, 15 W, Sankyo Denki G15T8E) or UV-C radiation (253 nm, UV Lamp, 15 W, Sankyo Denki G15T8). An overnight inoculum of the isolates, incubated at 28 °C, was diluted to an optical density at 600 nm (OD_600_) of 0.3, followed by a 1:10,000 additional dilution. Subsequently, 30 µL of the diluted sample was plated on R2A solid culture medium to obtain isolated colonies. UV-resistant bacteria were selected by exposing them to UV-B and UV-C doses of 38, 57, 76, and 113 J/m². The percentage of survival was determined by colony counting of irradiated and non-irradiated bacteria. The calculation of the irradiation emitted at the exposure point (E) was determined using the following Eq. [58]:

$$P=\frac{E2{\pi }^{2}DL}{2\alpha + sin\left(2\alpha \right)}$$

where: P: lamp output (W), E: irradiance (W/m^2^), D: distance (m) from the center of the lamp to the exposure point, L: length (m) of the lamp, 𝛼: half of the subtended angle from the lamp to the exposure point (so 𝛼 = L/2D).

### Growth curves of UV-resistant bacteria

To analyze the growth dynamics of UV-resistant bacteria, an overnight culture was initiated with an initial optical density at 600 nm (OD_600_) of 0.05 in R2A liquid culture medium. The bacterial cultures were then incubated at temperatures of 20 and 28 °C with constant shaking at 180 RPM. To monitor bacterial growth, 500 µL aliquots were extracted at regular intervals of 2 h, and the OD_600_ was measured to assess the growth progression.

### Biosynthesis and purification of QDs

The biosynthesis of QDs followed the procedure outlined by Bruna et al., 2019 [20]. A bacterial culture in the stationary phase was subjected to centrifugation for 10 min at 6,500 RPM and washed twice with distilled water (ddH_2_O). The bacterial cells were then resuspended in Borax-Citrate buffer at pH 9.3, adjusting the OD_600_ to 0.85. Subsequently, CdCl_2_ and cysteine were added at final concentrations of 0.1 mM and 1 mM, respectively. The incubation of cells took place at 20 °C for UV-resistant bacteria and 37 °C for *E. coli*, both under stirring, for durations of 8, 25, 50, and 75 min to achieve nanoparticles of varying sizes. For purification, cells were centrifuged for 3 min at 15,000 RPM, and the supernatant containing the nanoparticles was obtained and filtered through a 22 μm filter. To eliminate Cd_2_^+^ ions and other small molecules, the solution containing the nanoparticles underwent centrifugation for 10 min at 4000 RPM using a 3 kDa Amicon® Ultra filter (Merck Millipore, Burlington, VT, USA).

### Spectroscopic characterization of QDs

Spectroscopic analysis of the QDs synthesized by bacteria was conducted using a microplate reader, SynergyTM H1 (BioTek Instrument Inc.). Absorbance spectra were determined within the range of 300 to 500 nm, and fluorescence emission spectra were measured between 400 and 700 nm with an excitation wavelength of 360 nm. The Full Width at Half Maximum (FWHM) of the fluorescence emission spectra was determined following established protocols [59]. The Band Gap of the QDs was determined using the Tauc ratio method, with a reference value of 4.13 eV at 300 nm. This involved identifying the absorbance peak value on the X-axis corresponding to the wavelength values in electron volts (eV) [16]. Quantum Yield (QY) was calculated by measuring the absorbances between 0.01 and 0.1 of the QDs with an excitation wavelength of 360 nm. The QY was determined using the following equation:

$$Q={Q}_{R}\frac{I}{{I}_{R}}\frac{O{D}_{R}}{OD}\frac{{n}^{2}}{{{n}^{2}}_{R}}$$

Where:

- Q: Quantum Yield.
- Q_R_​: Quantum Yield of the reference fluorophore Fluorescein (QY = 0.9).
- I: Integrated fluorescence intensity of the QDs.
- I_R_​: Integrated fluorescence intensity of the reference fluorophore Fluorescein.
- OD: Absorbance (ranging from 0.01 to 0.1).
- OD_R_​: Absorbance of the reference fluorophore Fluorescein.
- n: Solvent refractive index of QDs dissolved in water (*n* = 1.333).
- n_R_​: Solvent refractive index of the reference fluorophore Fluorescein dissolved in ethanol (*n* = 1.335).

### Structural and chemical characterization of QDs

The structural and chemical properties of the QDs were thoroughly investigated through various characterization techniques.

#### Dynamic light scattering (DLS) and transmission electron microscopy (TEM)

The size of the QDs was determined employing DLS and TEM. DLS measurements, performed in triplicate, involved taking a 1 mL aliquot of QDs biosynthesized at 8, 25, 50, and 75 min, utilizing a Zetasizer Nano (ZS) from Malvern Instruments Ltd. For TEM, micrographs of purified QDs were acquired using a Philips Tecnai 12 BioTwin microscope at 80 kV. QD sizes were analyzed with Fiji-ImageJ software and presented through size-frequency histograms.

#### Chemical characterization by fourier transform infrared spectroscopy (FT-IR)

The purified QDs underwent chemical analysis using Fourier Transform Infrared Spectroscopy. FT-IR spectra were obtained with a Nicolet™ iSTM10 FT-IR Spectrometer (Thermo Scientific Inc.) equipped with a Smart iTRTM Attenuated Total Reflectance (ATR) accessory featuring a single-bounce Ge crystal. The scan frequency ranged from 4000 to 500 cm−¹, allowing for the identification of chemical functional groups and providing insights into the molecular composition of the QDs.

### QD photostability test

To assess the impact of UV-B radiation on the biosynthesized CdS QDs, a 1 mL aliquot of purified QDs with green, yellow, and red fluorescence emissions was subjected to UV-B radiation (UV Lamp, 15 W, Sankyo Denki G15T8) for durations of 10–20 min. Following exposure, the fluorescence spectrum of CdS QDs was measured in the range of 400–700 nm, employing a multiplate reader SynergyTM H1 from BioTek Instrument Inc. The excitation wavelength used was 360 nm. The fluorescence percentage was determined by evaluating the decay of the fluorescence peak in the irradiated QDs in comparison to non-irradiated QDs.

### Methylene blue degradation assay

Photodegradation experiments were conducted to assess the degradation efficiency of methylene blue in the presence of purified QDs at a concentration of 10 mg/mL. A methylene blue solution with a concentration of 10 mg/L was prepared and combined with the QDs. The photodegradation process was initiated by exposing the methylene blue-QD solution to UV-B radiation within the range of 280–360 nm. Various exposure durations, specifically 30, 60, and 90 min, were employed to evaluate the time-dependent effects on degradation. To quantify the extent of methylene blue degradation, absorbance measurements were taken at 664 nm using a SynergyTM H1 multi-plate reader (BioTek Instrument Inc.). The percentage of degradation was calculated based on the reduction in absorbance, providing insights into the photostability and potential photocatalytic activity of the synthesized QDs in the degradation of methylene blue.

### Electronic supplementary material

Below is the link to the electronic supplementary material.

Supplementary Material 1

Supplementary Material 2

Supplementary Material 3

Supplementary Material 4

## Data Availability

The datasets generated during and/or analyzed during the current study are available from the corresponding author on reasonable request.

## Abbreviations

DLS: Dynamic Light Scattering
EDS: Energy-dispersive X-ray spectroscopy
FTIR: Fourier-Transform Infrared
FWHM: Full Width at Half Maximum
^·^OH: Hydroxyl radical
O_2_: Molecular oxygen
PFD: Photoinduced Fluorescence Enhancement
H^+^: Positively Charged Hole
QDs: Quantum Dots
QY: Quantum Yield
ROS: Reactive Oxygen Species
^·^O_2_^−^: Superoxide radical
TEM: Transmission Electron Microscopy
XRD: X-ray diffraction
