# Supplementary material for: Biosynthesis of photostable CdS quantum dots by UV-resistant psychrotolerant bacteria isolated from Union Glacier, Antarctica
Source: Microb Cell Fact. 2024 May 17;23:140. doi: 10.1186/s12934-024-02417-x (PMC11100238; doi:10.1186/s12934-024-02417-x)
Supplement: Supplementary file 1 — Supplementary Material 1 [file 12934_2024_2417_MOESM1_ESM.docx]

**
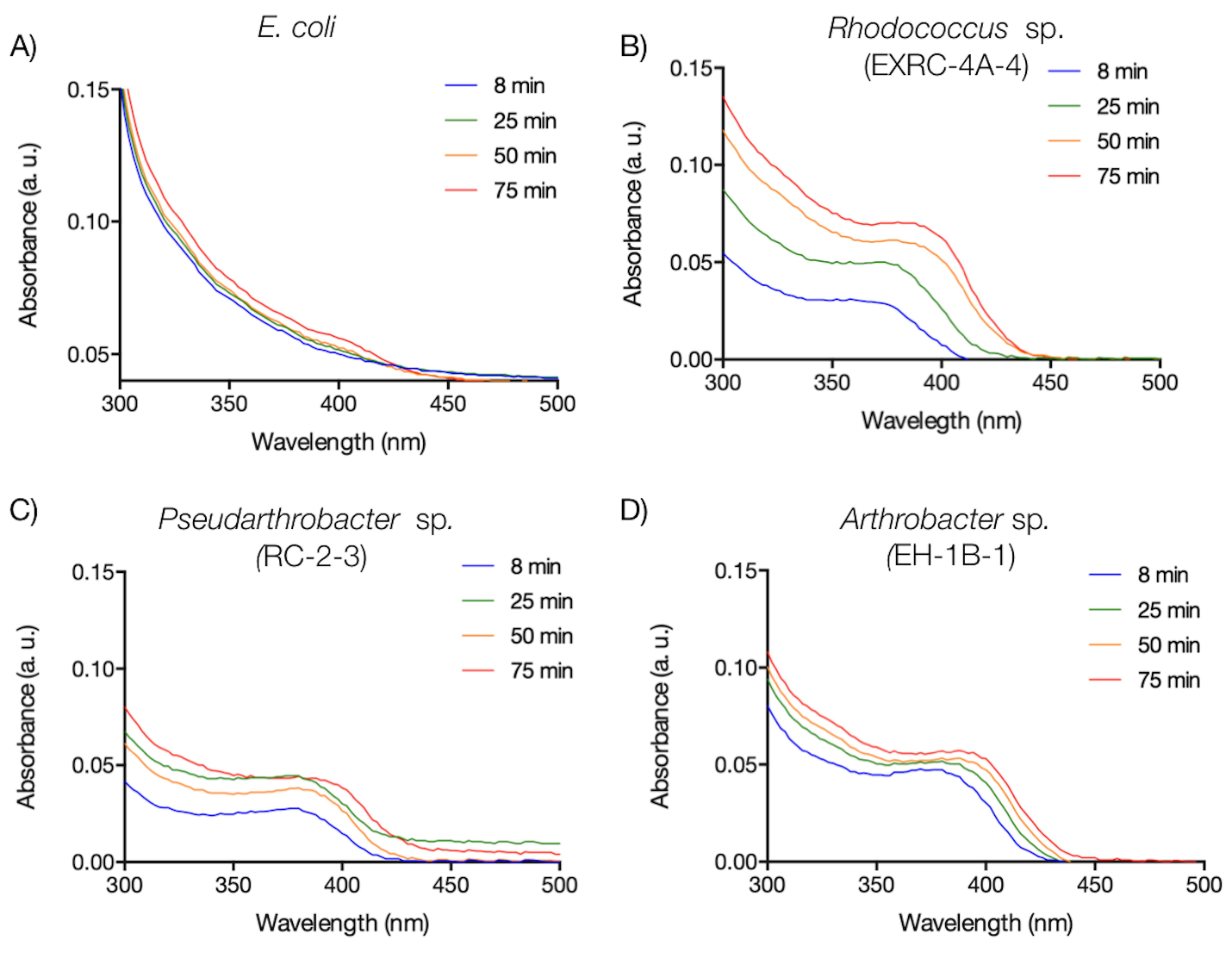
**

**Supplementary Figure 1. Absorbance spectra of QDs biosynthesized by UV-resistant bacteria.** Absorbance spectra of QDs biosynthesized at 20 °C was measured from 300 to 500 nm. QDs produced by *E. coli* (A), *Rhodococcus* sp. (EX-RC-4A-4) (B), *Pseudarthrobacter* sp. (RC-2-3) (C), and *Arthrobacter* sp. (EH-1B-1) (D).
