# Supplementary material for: Biosynthesis of photostable CdS quantum dots by UV-resistant psychrotolerant bacteria isolated from Union Glacier, Antarctica
Source: Microb Cell Fact. 2024 May 17;23:140. doi: 10.1186/s12934-024-02417-x (PMC11100238; doi:10.1186/s12934-024-02417-x)
Supplement: Supplementary file 2 — Supplementary Material 2 [file 12934_2024_2417_MOESM2_ESM.docx]

**
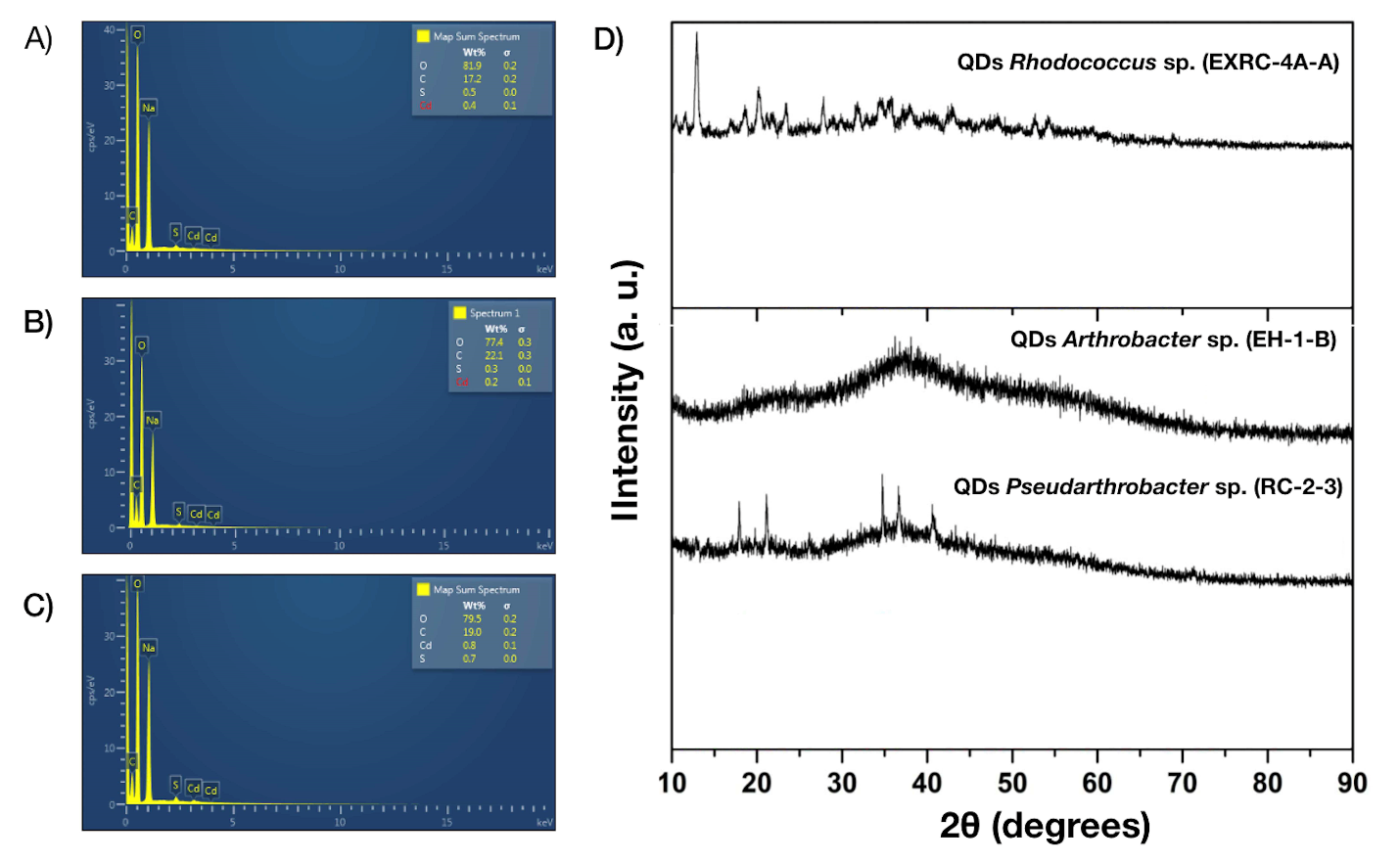
**

**Supplementary Figure 2.** EDS and XRD analysis of QDs biosynthesized by UV-resistant bacteria. EDS analysis of *Rhodococcus* sp. (EX-RC-4A-4) (A), *Arthrobacter* sp. (EH-1B-1) (B), *Pseudarthrobacter* sp. (RC-2-3) (C), and XRD analysis of QDs synthesized by UV-resistant bacteria isolated from Union Glacier (D).
