# Supplementary material for: Biosynthesis of photostable CdS quantum dots by UV-resistant psychrotolerant bacteria isolated from Union Glacier, Antarctica
Source: Microb Cell Fact. 2024 May 17;23:140. doi: 10.1186/s12934-024-02417-x (PMC11100238; doi:10.1186/s12934-024-02417-x)
Supplement: Supplementary file 3 — Supplementary Material 3 [file 12934_2024_2417_MOESM3_ESM.docx]

**
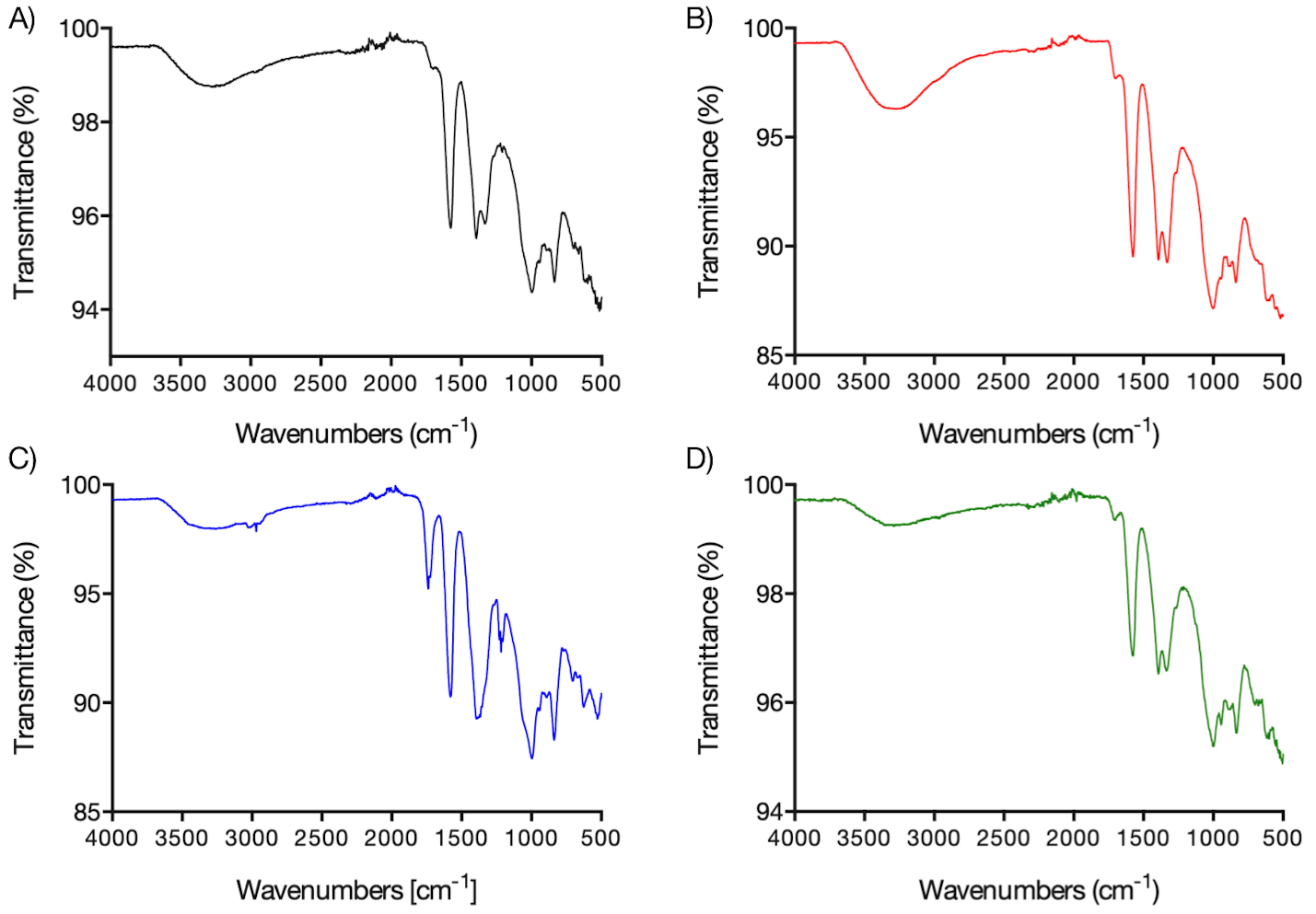
**

**Supplementary Figure 3. FTIR analysis of CdS QDs biosynthesized by UV-resistant bacteria.** FTIR spectra of *E. coli* (A), *Rhodococcus* sp. (EX-RC-4A-4) (B), *Pseudarthrobacter* sp. (RC-2-3) (C), and *Arthrobacter* sp. (EH-1B-1) (D).
