# Supplementary material for: Biosynthesis of photostable CdS quantum dots by UV-resistant psychrotolerant bacteria isolated from Union Glacier, Antarctica
Source: Microb Cell Fact. 2024 May 17;23:140. doi: 10.1186/s12934-024-02417-x (PMC11100238; doi:10.1186/s12934-024-02417-x)
Supplement: Supplementary file 4 — Supplementary Material 4 [file 12934_2024_2417_MOESM4_ESM.docx]

**
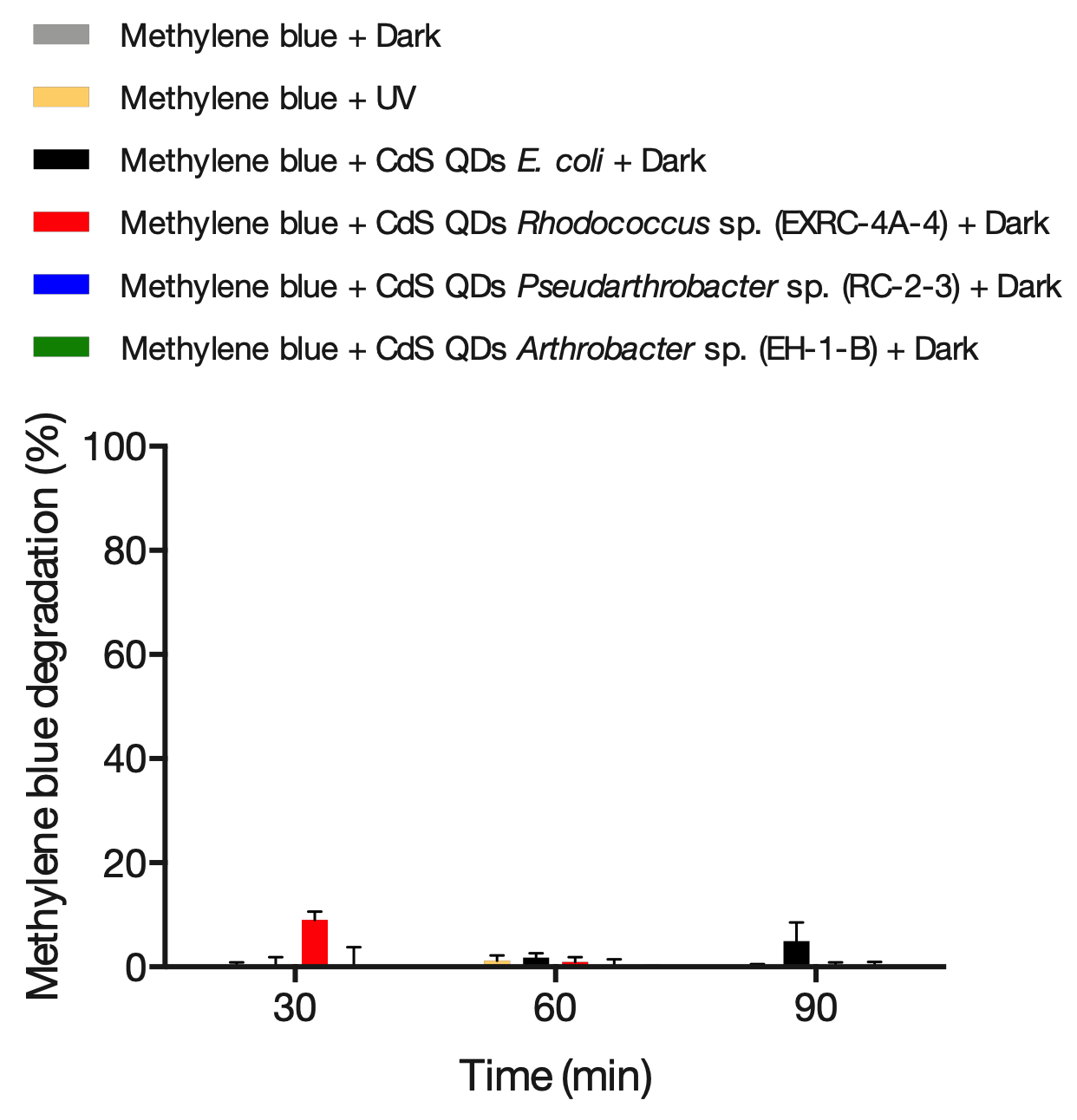
**

**Supplementary Figure 4. Methylene blue degradation under control conditions.** Methylene blue degradation was evaluated in light and dark conditions, and in the presence of non-irradiated QDs produced by *E. coli*, *Rhodococcus* sp. (EXRC-4A-4), *Pseudarthrobacter* sp. (RC-2-3), and *Arthrobacter* sp. (EH-1B-1).
